# The Late Dr. W. Lewis Thomson

**Published:** 1915-04-10

**Authors:** 


### The Late Dr. W. Lewis Thomson.

AN APPRECIATION.

This Easter has been saddened for many Derbyshire people by the death of Dr. W. Lewis Thomson, assistant county medical officer.

Dr. Thomson, who graduated M.D. at Glasgow University, was school medical officer, and had charge of the *x*-ray apparatus at the Derby Clinic and of the Tuberculosis Dispensary. He was lately appointed medical adviser to the Mental Deficiency Act Committee. It is interesting to recall that at the Buxton Congress of the Royal Institute of Public Health in 1908 Dr. Thomson read a paper on 208 cases of epidemic cerebro-spinal meningitis which he had observed in Lanarkshire.

It is thought that the doctor contracted the short illness of which he died while conducting bacteriological investigations for the troops stationed in South Derbyshire and South Notts. He was buried at The Mumbles, Glamorgan, beside the sea, in the beautiful county he loved, among the friends who knew him before he came to Derby. Dr. Thomson was forty years of age, and leaves a widow and three little children (as yet too young to realise the greatness of their loss).

Dr. Thomson was a first-class bacteriologist, a recognised authority on cerebro-spinal meningitis, an *x*-ray expert, and was experienced in tuberculosis work. His scientific and professional attainments make his loss a great one to the profession, as well as to his personal friends.

His modesty and unselfishness have prevented the full recognition of his worth, but those whom he has helped, those who have known him, must needs be better men and women for his brave, unselfish life.

